# Standard care informed by the result of a placental growth factor blood test versus standard care alone in women with reduced fetal movement at or after 36^+0^ weeks’ gestation: a pilot randomised controlled trial

**DOI:** 10.1186/s40814-020-0561-z

**Published:** 2020-02-13

**Authors:** Lindsay Armstrong-Buisseret, Peter J. Godolphin, Lucy Bradshaw, Eleanor Mitchell, Sam Ratcliffe, Claire Storey, Alexander E. P. Heazell

**Affiliations:** 1 Nottingham Clinical Trials Unit (NCTU), Building 42, University of Nottingham, University Park, Nottingham, NG7 2RD UK; 2 Maternal and Fetal Health Research Centre, 5th Floor (Research), St Mary’s Hospital, Oxford Road, Manchester, M13 9WL UK; 3 International Stillbirth Alliance, c/o Maternal and Fetal Health Research Centre, 5th Floor (Research,), St Mary’s Hospital, Oxford Road, Manchester, M13 9WL UK; 4 St. Mary’s Hospital, Manchester University Hospitals NHS Foundation Trust, Manchester Academic Health Science Centre, Manchester, M13 9WL UK

**Keywords:** Reduced fetal movement, Placental dysfunction, Placental biomarker, sFlt-1/PlGF ratio, Adverse pregnancy outcome, Feasibility study

## Abstract

**Background:**

Biomarkers of placental function can potentially aid the diagnosis and prediction of pregnancy complications. This randomised controlled pilot trial assessed whether for women with reduced fetal movement (RFM), intervention directed by the measurement of a placental biomarker in addition to standard care was feasible and improved pregnancy outcome compared with standard care alone.

**Methods:**

Women aged 16–50 years presenting at eight UK maternity units with RFM between 36^+0^ and 41^+0^ weeks’ gestation with a viable singleton pregnancy and no indication for immediate delivery were eligible. Participants were randomised 1:1 in an unblinded manner to standard care and a biomarker blood test result revealed and acted on (intervention arm) or standard care where the biomarker result was not available (control arm). The objectives were to determine the feasibility of a main trial by recruiting 175–225 participants over 9 months and to provide proof of concept that informing care by measurement of placental biomarkers may improve outcome. Feasibility was assessed via the number of potentially eligible women, number recruited, reasons for non-recruitment and compliance. Proof of concept outcomes included the rates of the induction of labour and caesarean birth, and a composite adverse pregnancy outcome.

**Results:**

Overall, 2917 women presented with RFM ≥ 36 weeks, 352 were approached to participate and 216 (61%) were randomised (intervention *n* = 109, control *n* = 107). The main reason for not approaching women was resource/staff issues (*n* = 1510). Ninety-seven women declined the trial, mainly due to not liking blood tests (*n* = 24) or not wanting to be in a trial (*n* = 21). Compliance with the trial interventions was 100% in both arms. Labour was induced in 97 (45%) participants (intervention *n* = 49, control *n* = 48), while 17 (9%) had planned caesarean sections (intervention *n* = 9, control *n* = 8). Overall, 9 (8%) babies in the intervention arm had the composite adverse pregnancy outcome versus 4 (4%) in the control arm.

**Conclusions:**

A main trial using a placental biomarker in combination with delivery, as indicated by the biomarker, in women with RFM is feasible. The frequency of adverse outcomes in this population is low, hence, a large sample size would be required along with consideration of the most appropriate outcome measures.

**Trial registration:**

ISRCTN, ISRCTN12067514; registered 8 September 2017.

## Introduction

In 2015, the stillbirth rate in the UK ranked 24th out of 49 high-income countries [1] and while the rate has declined over recent years [2], further reduction is a national priority [3]. Maternal perception of reduced fetal movements (RFM) is associated with stillbirth [3–7] and is thought to be a symptom of placental dysfunction restricting the supply of nutrients or oxygen to the fetus [8, 9]. A systematic review of management for RFM concluded that there was insufficient evidence to guide practice and that high-quality trials were required [10]. Furthermore, the need for evidence is supported by the two UK Confidential Enquiries into perinatal death which have identified management of RFM as a key area for action [11, 12].

The Stillbirth Priority Setting Partnership [13] identified two priorities relevant to RFM and placental dysfunction: (i) “which investigations identify a fetus at risk of stillbirth after a mother has experienced RFM” and (ii) “how can the structure and function of the placenta be assessed during pregnancy to detect potential problems and reduce the risk of stillbirth?” Research into this area also needs to balance the possibility of increasing perinatal morbidity and mortality by intervening to deliver babies too early versus the increased risk of stillbirth at later gestational ages [14]. A systematic review identified three studies including 3475 participants to assess the effectiveness of measuring placental biomarkers to improve pregnancy outcome and concluded there was insufficient evidence to draw any conclusions [15]. Therefore, studies to evaluate the potential benefit of assessing placental dysfunction via a novel biomarker in combination with delivery, as indicated by the biomarker, in women with RFM at or after 36 weeks’ gestation are warranted.

The multicentre randomised controlled Reduced Fetal Movement Intervention-2 (ReMIT-2) pilot trial reported here was based on the design of a single-centre randomised controlled feasibility trial (ReMIT) which investigated the intensive management of RFM via ultrasound scan and measurement of human placental lactogen (hPL) as a biomarker of placental dysfunction versus standard care [16]. The results showed that this type of trial was feasible with good compliance to the trial intervention (100% in both arms) and a potential improvement in proof of concept outcomes [16]. However, during the design stage of ReMIT-2, we evaluated whether prospective sites throughout the UK could test for hPL and determined this would not be viable in a multicentre trial due to the resource-intensive nature of the assay and the need for results to be available within 24 h.

A recent systematic review of diagnostic test accuracy studies evaluated the ability of placental biomarkers to detect pregnancies ending in the birth of a small for gestational age (SGA) infant or a stillbirth compared with those identified via the ultrasound assessment of estimated fetal weight (EFW). This review determined that placental growth factor (PlGF) gave the highest diagnostic odds ratio (49.2; 95% confidence interval [CI] 12.7 to 191) for detecting pregnancies ending in stillbirth and performed well in the prediction of SGA neonates [17]. As the data were based on 5894 pregnancies of which only 16 ended in stillbirth and no studies investigated EFW in the prediction of stillbirth, the review concluded that further research was required to determine the diagnostic accuracy of placental biomarkers alone and in combination with EFW in the identification of adverse pregnancy outcomes [17]. In addition, a cohort study of 300 women with RFM after 28 weeks’ gestation found that the addition of PlGF measurement improved the sensitivity for the detection of a composite adverse pregnancy outcome to 36% from 19% [18]. Furthermore, automated PlGF assays are available which are quick and easy to perform, making this a more viable option for a multicentre trial and clinical practice. Thus, PlGF was selected as the biomarker for further evaluation using a composite adverse pregnancy outcome in ReMIT-2.

PlGF is bound in maternal blood by soluble fms-like tyrosine kinase-1 (sFlt-1) [19], thus, assays to quantify PlGF often measure the sFlt-1/PlGF ratio. Currently, the sFlt-1/PlGF ratio of 38 is advocated by the National Institute for Health and Care Excellence as an aid in diagnosing preeclampsia in conjunction with other clinical information [20–22]. A diagnostic test accuracy study conducted in 289 women with RFM and an appropriately grown fetus showed that a sFlt-1/PlGF ratio of ≥ 38 had a sensitivity of 0.20 (95% CI 0.07 to 0.41) and a specificity of 0.88 (95% CI 0.83 to 0.92) to identify a composite adverse pregnancy outcome (perinatal death, birthweight < 5th centile, umbilical cord pH < 7.1 or admission to neonatal intensive care unit (NICU) for > 48 h) [23]. Although modest, this level of sensitivity was comparable to, or better than, other methods currently used to assess RFM, e.g. ultrasound fetal biometry, liquor volume and umbilical artery Doppler [23]. Views on this level of test accuracy were sought from the patient and public involvement (PPI) group and an independent Trial Steering Committee. Both agreed that the addition of sFlt-1/PlGF testing to currently available regimes, without a significant reduction in specificity, may aid the clinical management of women at risk of an adverse pregnancy outcome and was deemed an appropriate test to investigate further in this pilot trial.

The ReMIT-2 trial described here was conducted as a pilot study to assess the feasibility of a large main trial and to provide initial proof of concept that informing care by measurement of the sFlt-1/PlGF ratio may improve neonatal outcomes.

## Methods

The reporting of this trial follows the Consolidated Standards of Reporting Trials (CONSORT) statement extension to randomised pilot and feasibility trials recommendations (Additional file 1) [24].

### Design

This was a multicentre, randomised (1:1) controlled pilot trial of standard care informed by the results of an additional blood test for the sFlt-1/PlGF ratio versus standard care in women presenting with RFM at or after 36^+0^ weeks’ gestation. The trial was conducted at eight UK maternity units (detailed in Additional file 2) and the protocol was published prior to study completion [25].

### Participants

Women were eligible if they presented with RFM before the onset of labour between 36^+0^ and 41^+0^ weeks’ gestation (assessment of gestation was based upon the first trimester dating scan), had a viable singleton pregnancy with no indication for immediate delivery and provided written informed consent. Exclusion criteria were maternal age < 16 years or > 50 years, a fetus known to have any congenital anomalies as per the Fetal Anomalies Screening Programme (FASP) [26], multiple pregnancy, women for whom it was their first attendance to any antenatal care, previous randomisation into the ReMIT-2 trial and concurrent participation in the intervention phase of another clinical trial which determined the timing or mode of delivery.

All participants were contacted for follow-up approximately 6 weeks after birth and asked to complete a Postnatal Questionnaire which was sent in the post. The questionnaire consisted of the SF-12™ Health Survey, the Generalised Anxiety Disorder 2 (GAD-2) scale [27], participant views on the trial and health resource use details.

Women who declined to take part were asked if they were willing to complete an anonymous survey about their reasons for not participating in the trial. At the Chief Investigator’s site only, this sub-group was also asked if they were willing to have a short interview to further explore their reasons for not participating.

### Trial intervention

A blood sample was taken from all participants to measure the sFlt-1/PlGF ratio using the Elecsys® sFlt-1 and Elecsys® PlGF immunoassays (Roche Diagnostics; Germany). Participants were randomised 1:1 to standard care with either the blood sample tested locally at the time and the results revealed and acted on (intervention arm), or for the blood sample to be tested at a later time by a central laboratory so the result was not available and could not be acted on (control arm). Participants in the intervention arm with a sFlt-1/PlGF ratio ≥ 38 were offered delivery from 37^+0^ weeks by the most appropriate method with induction of labour (IOL) ideally commencing within 48 h of the offer. Those in the intervention arm with a sFlt-1/PlGF ratio < 38 or those in the control arm continued with usual care [4]. Participants in both arms were free to decline the recommended management plan and could return for any further episodes of RFM prior to delivery.

Central analysis of all blood samples, i.e. from both the intervention and control arms, was conducted using the same Elecsys® sFlt-1 and Elecsys® PlGF immunoassays (Roche Diagnostics; Germany) as used locally by each site. This was to provide a measure of reliability for the sFlt-1/PlGF ratio test and ensure that the results were consistent irrespective of where the assays were performed. The central analysis of sFlt-1 and PlGF was done in batches on a 6 monthly basis due to potential stability issues. The samples were also analysed centrally for hPL and other exploratory biomarkers as potential candidate markers of placental dysfunction to compare their diagnostic accuracy versus the sFlt-1/PlGF ratio test (results will be reported separately).

### Outcome measures

The main outcome was to determine the feasibility of a large-scale trial by aiming to recruit 175–225 participants over a period of 9 months, and associated outcome measures included number of potentially eligible women, number of women recruited at each site, proportion lost to follow-up and reasons for loss to follow-up, spectrum of clinical characteristics of women at randomisation, reasons for non-recruitment, compliance with the trial interventions and reasons for non-compliance and completeness of data collection for planned outcomes in a main trial. The thresholds for most of these feasibility outcomes were not specified as it was anticipated that results from this external pilot trial would inform any changes required before proceeding to a main trial. Additional information on feasibility describing participants’ and health professionals’ views and experiences of the trial will be reported separately.

Proof of concept outcomes for the mother included frequency of IOL or planned caesarean section and reasons for these procedures, frequency of maternal hypertensive disorders defined as the development of gestational hypertension or preeclampsia, maternal deaths prior to discharge or admissions to the intensive care unit (ICU). Proof of concept outcomes for the baby included stillbirths and deaths before discharge, 5-min Apgar score of < 7, umbilical artery pH < 7.05 and admission to the neonatal unit for > 48 h (these four components also formed the composite adverse pregnancy measure proposed as the primary outcome for a main trial at the time of designing ReMIT-2); SGA (< 10th centile on neonatal birthweight standards [28–30]); use of therapeutic cooling; length of stay in hospital; duration of respiratory support; and number of dependency days on the neonatal unit. For calculating the Gestation Related Optimal Weight (GROW) birthweight standard, the woman’s ethnicity was used with an ethnicity of white being classified as British European. In addition, the diagnostic performance of the placental factor test in participants allocated to the control arm only was included as a proof of concept outcome. The test results for participants in the intervention arm were not included in this outcome since the management of those participants could have been affected by the sFlt-1/PlGF result, potentially biasing the diagnostic performance outcome.

The impact on quality of life and resource use was assessed by the SF-12™ Health Survey [31] and a Health Resource Use Questionnaire and these results, along with the change in GAD-2 scale [27], will be reported separately.

### Sample size

As this was a feasibility trial, a formal sample size calculation for a between-group comparison was not appropriate. The target was to recruit 175–225 participants over 9 months from approximately 6 sites. This number would give estimated margins of error (half width of 95% CI) for the proportion recruited of approximately 5% and for the proportion lost to follow-up after discharge of approximately 7.5%.

### Randomisation

Eligible participants were randomised 1:1 to either the intervention or the control arm. Randomisation was stratified by site and number of weeks gestation when the participant first presented at hospital (< 40 weeks’ gestation or ≥ 40 weeks’ gestation). The randomisation schedule was based on a computer-generated pseudo-random code using random permuted blocks of randomly varying size, created by the Nottingham Clinical Trials Unit (NCTU) and held on a secure University of Nottingham server.

Investigators and delegated site staff randomised participants using an online randomisation system via a secure website developed and maintained by NCTU. It was not possible to blind participants or site staff to the allocated arm since those randomised to the intervention arm had the sFlt-1/PlGF ratio blood sample tested at the time and their results revealed to inform the next steps of their management plan.

### Statistical analysis

No formal statistical testing was conducted as the feasibility aims were to assess recruitment rates, proportion lost to follow-up and clinical characteristics in the target population. Descriptive statistics were used to summarise these results with the mean, standard deviation (SD) and/or median, minimum and maximum observations being reported for continuous variables while frequency counts and percentages were used for categorical variables. 95% CIs were calculated for the proportion of women recruited and the proportion of women lost to follow-up.

The proposed adverse pregnancy outcome for a main trial was summarised by allocated arm and the difference between arms presented as a relative risk and risk difference with 95% CIs. Of the four components of the adverse outcome, umbilical artery pH is often not measured in babies who are otherwise apparently healthy at birth, i.e. with an Apgar score at 5 min ≥ 7, particularly as higher Apgar scores are associated with less acidic umbilical artery pH values [32, 33]. Thus, for babies with missing umbilical artery pH data, a result ≥ 7.05 was assumed. We planned to report the estimates adjusted for the randomisation stratification variables, however, the models failed to converge so unadjusted estimates are given.

A scatter plot was used to compare sFlt-1/PlGF ratio results obtained from the central lab with those from each site. Agreement between the central lab and site results around the cut-off level of 38 was determined using unweighted kappa. A receiver operator characteristic (ROC) curve was plotted for the sFlt-1/PlGF ratio and adverse pregnancy outcome to determine the diagnostic performance of the biomarker test for participants in the control arm. All analyses were carried out using Stata® SE 15.1 (StataCorp LP, College Station, TX, USA).

## Results

**Fig. 1:**
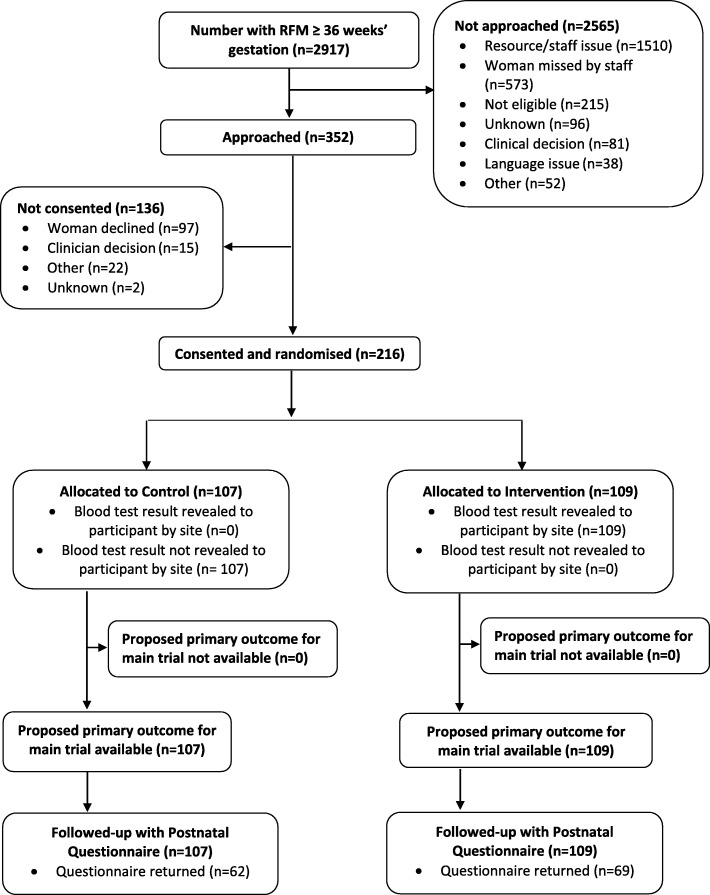
Flow of participants through the trial

Recruitment to the trial started in March 2018 and finished as planned in December 2018; follow-up of participants was completed in April 2019. A total of 2917 women presented with RFM ≥ 36 weeks’ gestation during the recruitment period (Fig. 1) and the numbers of potentially eligible women at each site ranged from 143–595 (Additional file 2). Of these, 352 (12%) were approached and 216 gave consent and were randomised into the trial (Fig. 1) which represented 7% of those presenting with RFM (95% CI 6.5 to 8.4%) and 61% of those approached (95% CI 56.2 to 66.3%). The number of women recruited at each site ranged from 12–58 (Additional file 2). A total of 85 (39%; 95% CI 33 to 46%) participants (40 in the intervention arm, 45 in the control arm) were lost to follow-up, all of whom did not return the Postnatal Questionnaire despite two reminders being sent (Fig. 1).

The main reasons for not approaching potentially eligible women were resource or staff issues (including women who presented out of hours; *n* = 1510) and women missed by trial staff (including women missed within normal working hours; *n* = 573; Fig. 1). Of the 97 women who were approached and declined to join the trial, the main reasons given were that they did not like having blood tests (*n* = 24) or did not want to be in a research trial (*n* = 21). Only 2 women who declined did so because they did not think the blood test was reliable enough to predict complications later in their pregnancy. None of the women who declined wished to discuss their reasons further in a short interview.

### Baseline characteristics

The two allocated arms were similar at the trial entry for maternal baseline characteristics (Table 1). The mean age of participants in the trial was 29.8 years, just under half (47%) were in their first pregnancy and 2 had previously had a stillbirth. A total of 8 (4%) participants (5 in the intervention arm, 3 in the control arm) had a SGA fetus in their current pregnancy using the INTERGROWTH-21st birthweight standard [28]. A similar proportion of participants had previous obstetric complications (19 in the intervention arm, 23 in the control arm) with 7 having a SGA baby in a previous pregnancy (2 in the intervention arm, 5 in the control arm; Table 1). All participants had a normal cardiotocograph (CTG) at trial entry. Of the babies, 113 (52%) were male (61 in the intervention arm, 52 in the control arm) and mean gestational age at delivery was 39^+5^ weeks.

**Table 1:** Baseline characteristics

Characteristic	Control (*n* = 107)	Intervention (*n* = 109)	Total (*n* = 216)
Maternal age (years)			
Mean (SD)	29.2 (5.7)	30.4 (5.4)	29.8 (5.6)
Gestational age at randomisation (weeks)			
Mean (SD)	37.8 (1.2)	37.9 (1.4)	37.9 (1.3)
*Stratification variable*			
≥ 40^+0^	7 (7%)	10 (9%)	17 (8%)
Ethnicity			
White	93 (87%)	93 (85%)	186 (86%)
Black	1 (1%)	5 (5%)	6 (3%)
South Asian	7 (7%)	6 (6%)	13 (6%)
East Asian	3 (3%)	3 (3%)	6 (3%)
Mixed Race	1 (1%)	2 (2%)	3 (1%)
Others	2 (2%)	0 (–)	2 (1%)
BMI at booking visit			
Mean (SD)	26.8 (5.6)	27.4 (6.3)	27.1 (6.0)
Parity			
0	48 (45%)	53 (49%)	101 (47%)
1	37 (35%)	36 (33%)	73 (34%)
≥ 2	22 (20%)	20 (18%)	42 (19%)
Number of previous stillbirths			
1	1 (1%)	1 (1%)	2 (1%)
Obstetric history for previous pregnancies^1^			
Yes	23 (21%)	19 (17%)	42 (19%)
*Obstetric cholestasis*	0 (–)	4 (4%)	4 (2%)
*Placental abruption*	2 (2%)	1 (1%)	3 (1%)
*Preeclampsia*	5 (5%)	1 (1%)	6 (3%)
*SGA baby*	5 (5%)	2 (2%)	7 (3%)
*Other*	16 (15%)	14 (13%)	30 (14%)
Diagnoses in current pregnancy^1^			
Antepartum haemorrhage	0 (–)	1 (1%)	1 (< 1%)
Hypertension	1 (1%)	1 (1%)	2 (1%)
Obstetric cholestasis	0 (0%)	2 (2%)	2 (1%)
Other^2^	6 (6%)	4 (4%)	10 (5%)
Past medical history^1^	15 (14%)	12 (11%)	27 (13%)
Hypertension	4 (4%)	0 (–)	4 (2%)
Diabetes	2 (2%)	0 (–)	2 (1%)
Other^3^	10 (9%)	12 (11%)	22 (10%)
Estimated fetal weight from ultrasound scan (g)			
Mean (SD)	3255 (520.6)	3109 (500.1)	3181 (514.4)
*Centile usingINTERGROWTH-21stbirthweight standard*			
Mean (SD)	67.4 (24.7)	59.1 (25.8)	63.3 (25.6)
SGA fetus	3 (3%)	5 (5%)	8 (4%)
Prescribed medication at the trial entry^1^			
Aspirin	8 (7%)	7 (6%)	15 (7%)
Other^4^	10 (9%)	11 (10%)	21 (10%)
Substance misuse during this pregnancy			
Benzodiazepine	0 (–)	3 (3%)	3 (1%)
Cigarette smoking status during this pregnancy			
Currently smoking	5 (5%)	3 (3%)	8 (4%)
Stopped smoking in this pregnancy	5 (5%)	4 (4%)	9 (4%)
Not smoked in this pregnancy	97 (91%)	102 (94%)	199 (92%)
Duration women concerned about baby’s movements (h)			
0–24	76 (71%)	72 (66%)	148 (69%)
25–48	9 (8%)	18 (17%)	27 (13%)
> 48	22 (21%)	19 (17%)	41 (19%)
Absent fetal movements			
Yes	37 (35%)	51 (47%)	88 (41%)
No	70 (65%)	58 (53%)	128 (59%)

All data are *N* (%) unless indicated

^1^Categories are not mutually exclusive

^2^Others include anxiety and depression (*n* = 2), gestational diabetes, placenta praevia, medullary sponge kidney, hypothyroidism, gallstones/pancreatitis, type 1 diabetes, GBS, thrush and fibromyalgia (not mutually exclusive)

^3^Others include significant cardiac disease (*n* = 3), significant gastrointestinal problems (*n* = 3), significant mental health problems (*n* = 10), renal disease (*n* = 1) and thyroid disease (*n* = 7)

^4^Others include antidepressants (*n* = 14), benzodiazepines (*n* = 3) and low molecular weight heparin (*n* = 6)

### Compliance with allocated trial intervention

In both allocated arms, compliance with the trial intervention, i.e. the process for taking and testing the blood samples, was 100% (Table 2). All participants in the intervention arm had a blood sample taken which was analysed at site to obtain the sFlt-1/PlGF ratio and the result revealed to the participant. Those in the control arm all had a blood sample taken, none of which were analysed at the site and therefore results could not be revealed to the participant (Table 2).

**Table 2:** Compliance with allocated trial intervention

	Control (*n* = 107)	Intervention (*n* = 109)	
Trial blood sample taken	107 (100%)	109 (100%)	
Blood sample analysed at site			
Yes	0 (–)	109 (100%)	
No	107 (100%)	0 (–)	
sFlt-1/PlGF ratio obtained by site and revealed to participant	–	109 (100%)	
Test result (sFlt-1/PlGF ratio)	–		
Mean (SD)	–	21.1 (23.9)	
Median (25th, 75th centile)	–	14.8 (5.3, 27.8)	
Min, max	–	0.6, 151.1	
Expedited delivery offered		sFlt-1/PlGF ratio < 38 (*n* = 93)	sFlt-1/PlGF ratio ≥ 38 (*n* = 16)
Yes	9 (8%)	8^1^ (9%)	15 (94%)
No	98 (92%)	85 (91%)	1^2^ (6%)
Expedited delivery accepted			
Yes	9 (100%)	8 (100%)	12 (80%)
No	0 (–)	0 (–)	3^3^ (20%)

All data are *N* (%) unless indicated

^1^Reasons for offering expedited delivery in the intervention arm with sFlt-1/PlGF ratio < 38 include the following: participant wanted induction of labour even though not indicated (*n* = 2), clinician decision to deliver baby (*n* = 5) and participant offered induction for recurrent RFM (*n* = 1)

^2^Reasons for not offering expedited delivery in the intervention arm with sFlt-1/PlGF ≥ 38 include the following: clinician decision to continue pregnancy (*n* = 1)

^3^Reasons for not accepting expedited delivery in the intervention arm with sFlt-1/PlGF ≥ 38 include the following: would like delivery on midwife-led unit, feels well and baby at the time was moving well (*n* = 1); discussed results with husband and decided not to accept expedited delivery (*n* = 1); induction date booked in 1 weeks’ time, would like to stay with that plan (*n* = 1)

In the intervention arm, 15/16 participants (94%) with a sFlt-1/PlGF ratio ≥ 38 were offered expedited delivery as per the protocol (Table 2). One participant with a sFlt-1/PlGF ratio ≥ 38 was not offered expedited delivery due to the clinician recommending to continue the pregnancy. Of those in the intervention arm with a sFlt-1/PlGF ratio < 38, 8/93 (9%) were also offered expedited delivery although this was not indicated by the blood test result (Table 2). The reasons for this offer included the participant wanted IOL (*n* = 2), clinician decision to deliver the baby (*n* = 5) and participant experiencing recurrent RFM (*n* = 1). A total of 9 (8%) participants in the control arm were offered and accepted expedited delivery as part of standard care.

### Completeness of data collection for planned outcomes in a main trial

The completeness of data collection for the components of the composite adverse pregnancy outcome proposed for a main trial was 100% in both allocated trial arms for stillbirth or death before discharge, 5-min Apgar score and length of stay in the neonatal unit (Table 3). The data collection of the umbilical artery pH was lower with 66% completeness in the intervention arm and 58% completeness in the control arm. However, as all babies where this data was missing had a 5-min Apgar score ≥ 7, an umbilical artery pH ≥ 7.05 was assumed [32, 33], which gave 100% of participants with an assessable composite adverse pregnancy outcome (Table 3).

**Table 3:** Completeness of data collection for planned outcomes in a main trial

Outcome	Control (*n* = 107)	Intervention (*n* = 109)	Total (*n* = 216)
Completeness of each component of proposed primary outcome			
Stillbirth or death before discharge	107 (100%)	109 (100%)	216 (100%)
5-min Apgar score	107 (100%)	109 (100%)	216 (100%)
Umbilical artery pH	62 (58%)	72 (66%)	134 (62%)
Length of stay in neonatal unit	107 (100%)	109 (100%)	216 (100%)
Number of primary outcome components with complete data for each participant			
0	0 (–)	0 (–)	0 (–)
1	0 (–)	0 (–)	0 (–)
2	0 (–)	0 (–)	0 (–)
3	45 (42%)	37 (34%)	82 (38%)
4	62 (58%)	72 (66%)	134 (62%)
*Participants with assessable composite primary outcome*^*1*^	107 (100%)	109 (100%)	216 (100%)

^1^Primary outcome is assessable if all components are complete, or if any component is positive regardless of missing data, or if umbilical artery pH is missing and all other components are negative

For the proposed secondary outcomes for a main trial, completeness of data collection was 100% for all components in both allocated trial arms including birthweight, use of therapeutic cooling, length of stay in hospital (babies), duration of mechanical respiratory support, number of dependency days on the neonatal unit, mode of delivery, length of stay in the maternity unit (women), maternal mortality and admission to ICU (women).

### Maternal proof of concept outcomes

A total of 20 participants experienced pregnancy complications after the baseline visit (15 in the intervention arm, 5 in the control arm; Table 4). Of these, 3 (3%) in the intervention arm had hypertension compared with 2 (2%) in the control arm. The most common complication occurring after randomisation was the prelabour rupture of membranes affecting 9 (8%) in the intervention arm and 1 (1%) in the control arm.

**Table 4:** Maternal proof of concept outcomes

	Control (*n* = 107)	Intervention (*n* = 109)
Complications of pregnancy after baseline visit^1^	5 (5%)	15 (14%)
Antepartum haemorrhage	2 (2%)	2 (2%)
Hypertension in pregnancy	2 (2%)	3 (3%)
Obstetric cholestasis	2 (2%)	1 (1%)
Prelabour rupture of membranes	1 (1%)	9 (8%)
Other^2^	1 (1%)	3 (3%)
Onset of labour		
Spontaneous	49 (46%)	50 (46%)
Induced	48 (45%)	49 (45%)
Caesarean section	10 (9%)	10 (9%)
If caesarean section, grade		
Grade 1 (emergency)	2 (20%)	1 (10%)
Grade 4 (elective)	8 (80%)	9 (90%)
Reasons for induction^3^		
Recommended by sFlt-1/PlGF test result	0 (–)	13 (27%)
Gestational age > 41 weeks	8 (17%)	7 (14%)
Term (> 37 weeks) prelabour rupture of membranes > 24 h	3 (6%)	5 (10%)
Fetal growth restriction	2 (4%)	1 (2%)
Reduced fetal movements	29 (60%)	25 (51%)
Pregnancy-induced hypertension	0 (–)	1 (2%)
Preeclampsia	1 (2%)	0 (–)
Obstetric cholestasis	1 (2%)	2 (4%)
Gestational diabetes	1 (2%)	2 (4%)
Maternal request	2 (4%)	3 (6%)
Other	14 (29%)	5 (10%)
Reasons for elective caesarean^4^		
Recommended by sFlt-1/PlGF test result	0 (–)	1 (11%)
Previous caesarean section	4 (50%)	5 (56%)
Non-cephalic presentation	2 (25%)	3 (33%)
Presumed fetal compromise	1 (13%)	0 (–)
Maternal request	0 (–)	2 (22%)
Previous 3rd/4th degree tear	0 (–)	1 (11%)
Other	2 (25%)	3 (33%)
Mode of delivery		
Vaginal birth	72 (67%)	64 (59%)
Instrumental vaginal birth	14 (13%)	25 (23%)
Emergency caesarean section	13 (12%)	11 (10%)
Elective caesarean section	8 (7%)	9 (8%)

^1^Complications of pregnancy are not mutually exclusive

^2^Others include spontaneous rupture of membranes, viral meningitis, polyhydramnios and genital ulcer (microbiology confirmed HSV-1)

^3^Reasons for induction are not mutually exclusive; denominator is the number of participants who were induced

^4^Reasons for elective caesarean are not mutually exclusive; denominator is the number of participants who had an elective caesarean section

The frequency of IOL was the same in both allocated trial arms (45%), while the frequency of planned caesarean section was similar between arms (8% in the intervention arm, 7% in the control arm; Table 4). The main reason for IOL in both allocated trial arms was RFM (51% in the intervention arm, 60% in the control arm), while the main indication for a planned caesarean section was having had a previous caesarean section (56% in the intervention arm, 50% in the control arm; Table 4). For those in the intervention arm, delivery offered due to the sFlt-1/PlGF result was the reason given for 13/49 (27%) participants who had IOL but only 1/9 (11%) for those who had a planned caesarean section (Table 4).

There were no maternal admissions to the ICU or maternal deaths prior to discharge and the median length of participant stay in the maternity unit was 2 days (interquartile range 1 to 3).

### Neonatal proof of concept outcomes

There were no stillbirths or neonatal deaths before discharge, 2 babies (1 in the intervention arm, 1 in the control arm) had a 5-min Apgar score < 7 and a total of 8 babies (4 in each arm) were admitted to the neonatal unit for > 48 h (Table 5). Four babies (4%) in the intervention arm had an umbilical artery pH < 7.05 compared with no babies in the control arm (Table 5). Overall, 9 (8%) babies in the intervention arm had the composite adverse pregnancy outcome compared with 4 (4%) in the control arm (Table 5); relative risk 2.21 (95% CI 0.70, 6.96); risk difference 4.51% (95% CI − 1.78%, 10.8%).

**Table 5:** Neonatal proof of concept outcomes

	Control (*n* = 107)	Intervention (*n* = 109)
Composite adverse pregnancy outcome^1^	4 (4%)	9 (8%)
Stillbirth or death before discharge	0 (–)	0 (–)
5-min Apgar score < 7	1 (1%)	1 (1%)
Umbilical artery pH < 7.05	0 (–)	4 (4%)
Admission to neonatal unit for > 48 h	4 (4%)	4 (4%)
Other neonatal outcomes		
SGA baby (INTERGROWTH-21st birthweight standard)	2 (2%)	9 (8%)
SGA baby (GROW birthweight standard)	7 (7%)	15 (14%)
Length of stay in hospital (days)		
Median (25th, 75th centile)	1.2 (0.8, 2.2)	1.2 (0.7, 2.4)
Outcomes for babies admitted to neonatal unit	*n* = 5	*n* = 7
Use of therapeutic cooling	0 (-)	0 (–)
Duration of mechanical respiratory support		
< 24 h	0 (–)	2 (29%)
1–2 days	2 (40%)	1 (14%)
Length of stay in neonatal unit^2^		
Normal care	*n* = 0	*n* = 1
< 24 h	0 (–)	0 (–)
1–2 days	0 (–)	1 (14%)
> 2 days	0 (–)	0 (–)
Special care	*n* = 5	*n* = 6
<24 h	0 (–)	1 (14%)
1–2 days	2 (40%)	2 (29%)
> 2 days	3 (60%)	3 (43%)
High dependency care	*n* = 5	*n* = 2
< 24 h	0 (–)	1 (14%)
1–2 days	1 (20%)	1 (14%)
> 2 days	2 (40%)	0 (–)
Intensive care	*n* = 1	*n* = 1
< 24 h	0 (–)	1 (14%)
1–2 days	1 (20%)	0 (–)
> 2 days	0 (–)	0 (–)

All data are *N* (%) unless indicated

^1^This means at least one of stillbirth or death before discharge, 5-min Apgar score of < 7, umbilical artery pH < 7.05, or admission to the neonatal unit for > 48 h. If umbilical artery pH was missing (*n* = 82) and all other components did not indicate the composite adverse pregnancy outcome, an umbilical artery pH ≥ 7.05 was assumed for the purposes of calculating the composite

^2^Levels of care are not mutually exclusive

Using INTERGROWTH-21st [28] as the birthweight standard gave a total of 11 (5%) SGA babies (9 in the intervention arm, 2 in the control arm), whereas GROW [30] gave a total of 22 (10%) SGA babies (15 in the intervention arm, 7 in the control arm; Table 5). It was not possible to calculate SGA using the Ponderal Index as planned since the length of babies is no longer routinely collected at birth. Of the 22 SGA babies defined by GROW, 16 were delivered ≤ 39 weeks’ gestation and a greater proportion of these were in the intervention arm (12 in the intervention arm versus 4 in the control arm).

The median length of stay in the hospital for babies was 1.2 days (interquartile range 0.7 to 2.2). Overall, 12 babies were admitted to the neonatal unit (7 in the intervention arm, 5 in the control arm), none of whom required therapeutic cooling (Table 5). The number of dependency days on the neonatal unit varied with the level of care being received (Table 5). Of the 7 babies in the intervention arm admitted to the neonatal unit, 1 had a length of stay < 24 h, 2 were admitted for 1–2 days and 4 were on the neonatal unit for > 2 days. Of the 5 babies in the control arm admitted to the neonatal unit, 1 had a length of stay of 1–2 days and 4 were admitted for > 2 days. Mechanical respiratory support was provided for 5 babies (3 in the intervention arm, 2 in the control arm); 2 babies in the intervention arm required this respiratory support for < 24 h, while 1 baby in the intervention arm and 2 in the control arm needed respiratory support for 1–2 days (Table 5).

### Central lab analysis and diagnostic performance of sFlt-1 and PlGF

A comparison of sFlt-1/PlGF ratio results for intervention arm samples tested at both the sites and the central lab showed good agreement (central lab versus site, mean difference 0.46, SD 4.04; Additional file 3). Of the 93 samples with a sFlt-1/PlGF ratio < 38 tested at the sites, 92 were also < 38 in central lab testing. A total of 16 samples tested at sites had a sFlt-1/PlGF ratio ≥ 38, while the central lab reported 17 samples with this result which gave an unweighted kappa of 0.96.

**Fig. 2:**
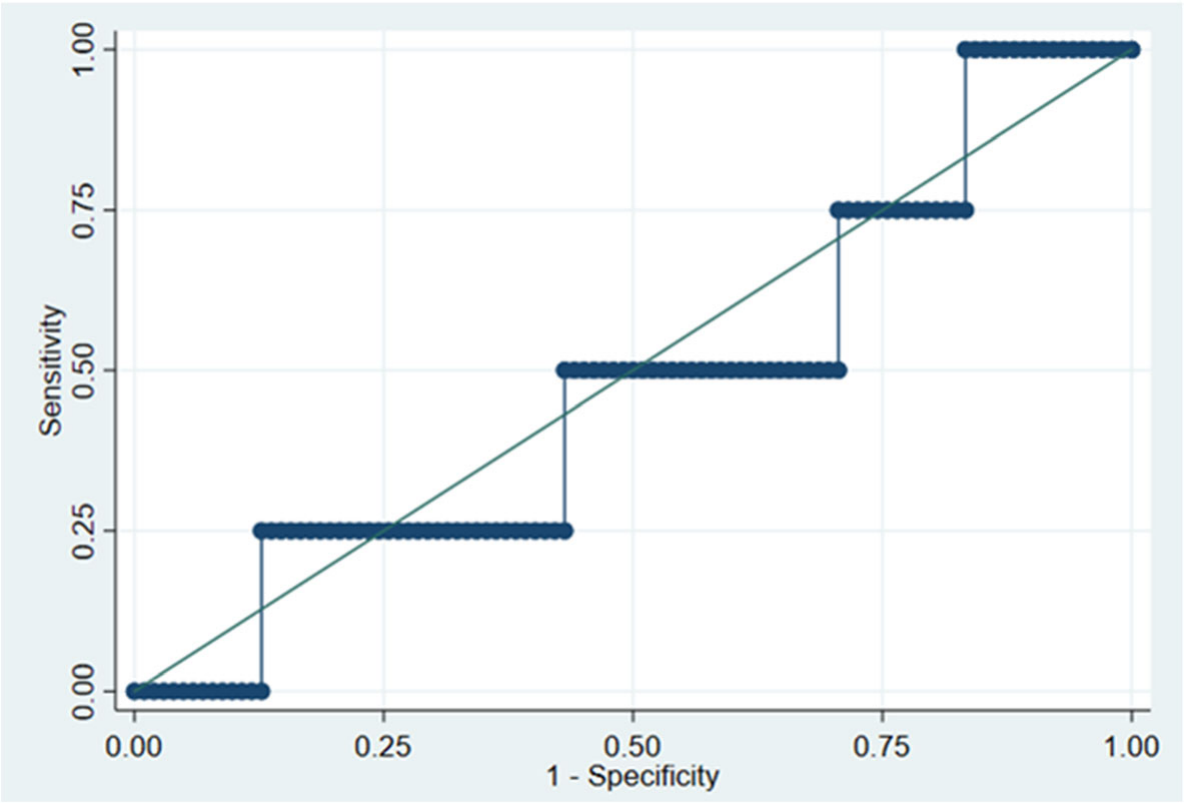
Receiver operator characteristic curve for sFlt-1/PlGF ratio and composite adverse pregnancy outcome in the control arm

For the 107 participants in the control arm, central lab analysis showed 88 (82%) had a sFlt-1/PlGF ratio result < 38, while 18 (17%) had a sFlt-1/PlGF ratio result ≥ 38. The blood sample for 1 participant was erroneously destroyed and could not be tested. Of the 88 participants with a sFlt-1/PlGF ratio < 38, 3 (3%) had the composite adverse pregnancy outcome, while 1/18 (6%) with a sFlt-1/PlGF ratio ≥ 38 had the composite which gave an area under the ROC (AUROC) curve of 0.48 (95% CI 0.16 to 0.79; Fig. 2) as a measure of the diagnostic performance in the control arm.

## Discussion

This randomised controlled pilot trial has provided important information which will help guide the design and conduct of a larger main trial in women with RFM. Sufficient numbers of eligible women were available and we were able to recruit to target. The length of time individual sites were open to recruitment varied with half the sites being open for only 5–6 months (Additional file 2) which is extremely encouraging for a main trial and suggests that recruitment could have been above target with a faster site set-up time.

The percentage of eligible women randomised varied across sites (41–83%; Additional file 2) from those who were approached. Resource or staff issues and potentially eligible women being missed were the main reasons for not approaching women and are limitations of this pilot trial. Due consideration would need to be given to strategies to overcome this in a future trial, potentially including the provision of out-of-hours resource to ensure that women presenting at any time of the day can be approached. Additional methods for raising awareness of the trial would also need to be developed as a reminder for trial staff.

The baseline characteristics of participants in both allocated trial arms were comparable to previous cohort and intervention studies of RFM [16, 18]. Compliance with the trial interventions was excellent for the blood testing process and adherence to the offer of expedited delivery for those in the intervention arm with a sFlt-1/PlGF ratio ≥ 38. A small number of participants in both the intervention arm with a sFlt-1/PlGF ratio < 38 and the control arm were also offered expedited delivery which was to be expected as part of standard care and may reflect other pregnancy complications which occurred after recruitment into the trial such as prelabour rupture of membranes or gestational hypertension.

Completeness of data collection was also excellent for the vast majority of the proposed primary and secondary outcome measures for a main trial. One of the limitations of this trial was collection of umbilical artery pH data, however, this is often not measured in babies who are otherwise healthy at birth, particularly as higher Apgar scores at 5 min are associated with less acidic umbilical artery pH values [32, 33]. Thus, any babies where this data was missing were assumed to have had an umbilical artery pH ≥ 7.05, especially as they all had a 5-min Apgar score ≥ 7. For a main trial, further consideration would need to be given to the use of appropriate and clinically important outcome measures that are routinely collected to ensure the results are robust and widely generalisable. Another limitation of the trial was the response rate for the Postnatal Questionnaire (131/216; 61%; Fig. 1) and in a main trial, options for increasing this could be included such as providing the opportunity to complete questionnaires online or text alerts sent before and after the questionnaire.

These results have built on the initial findings from the ReMIT trial [16] and further demonstrate that a main trial to assess placental function via a biomarker in maternal blood in combination with delivery, as indicated by the biomarker, is feasible in women with RFM.

There was no difference in IOL and planned caesarean section rates between the two allocated trial arms, and the frequency of IOL for RFM was very similar. Interestingly, the proportion of IOL recommended by the sFlt-1/PlGF ratio was comparable with the proportion having IOL based on the hPL result in the ReMIT trial (27% versus 30% [16];) although the numbers are too small to draw any conclusions and it is plausible that a similar proportion of cases had evidence of placental dysfunction.

Although a higher proportion of babies in the intervention arm had the composite adverse pregnancy outcome compared with the control arm (difference was due to 4 babies in the intervention arm having an umbilical artery pH > 7.05), the numbers are too small to draw any firm conclusions. Overall, the composite adverse pregnancy outcome occurred in 13/216 (6%) babies. In the first ReMIT trial, the comparable composite poor pregnancy outcome of stillbirth, admission to NICU or umbilical artery pH < 7.1 occurred in 8/120 (7%) babies; this increased to 24/120 (20%) babies when birthweight ≤ 10th centile was included in the composite [16]. Interestingly, of the SGA babies in ReMIT-2, a slightly greater proportion were delivered prior to 39 weeks’ gestation in the intervention arm than in the control arm suggesting that identification of SGA babies in the intervention arm was more likely, which then prompted delivery. This observation would be consistent with the findings of the review of diagnostic test accuracy studies [17]. Nevertheless, the use of a composite outcome is a limitation of the current trial and our findings reinforce the need to ensure appropriate outcome measures are used in a main trial to enable robust conclusions to be drawn. Consideration will be given to inclusion of birthweight ≤ 10 centile in the composite adverse pregnancy outcome for a main trial, particularly in light of the updated NHS Saving Babies’ Lives Care Bundle released in 2019 which includes management of babies at risk of fetal growth restriction as one of its key standards [3] and evidence that identification of a SGA fetus at term prior to labour is associated with improved outcome at birth [34].

Diagnostic performance of the sFlt-1/PlGF ratio in the control arm gave an AUROC of 0.48 (95% CI 0.16 to 0.79) however, as the number of participants with a composite adverse pregnancy outcome was so small, caution is advised in any interpretation of this result as the 95% CI is very wide. Encouragingly, of the 215 participants with a sFlt-1/PlGF ratio result, 34 (15.8%) were ≥ 38, which was similar to the proportion seen in the pre-trial diagnostic test accuracy work (12.8%) suggesting that the test is performing consistently in women with RFM [23].

Although most research utilising the sFlt-1/PlGF ratio in placental dysfunction has been targeted at preeclampsia [21], more recent work has been investigating the use of these biomarkers for detecting other pregnancy complications such as the identification of SGA babies [35–37], fetal compromise associated with RFM [18] and gestational hypertension and placental abruption [38]. It is likely that interest will increase in the use of biomarkers combined with other aspects of care for the management of RFM after results from the AFFIRM trial indicated that a care package for RFM did not significantly reduce the rate of stillbirth but increased the rate of obstetric intervention [39]. Further work to investigate the potential of placental biomarkers to aid prediction of adverse pregnancy outcomes in areas such as RFM is therefore warranted.

## Conclusions

We have demonstrated that a large main trial assessing placental dysfunction via a biomarker in combination with delivery, as indicated by the biomarker, in women with RFM ≥ 36 weeks’ gestation is feasible based on meeting the recruitment target and excellent compliance with the trial interventions. Some aspects of the design require modification, in particular further consideration will be given to selecting the most accurate biomarker and the most appropriate and clinically important outcome measures to ensure robust conclusions can be drawn. Results from an adequately powered main trial would help address key areas for action noted in the two perinatal Confidential Enquiries [11, 12] and some of the Stillbirth Priority Setting Partnership research priorities, notably “Can the wider use of existing tests and monitoring procedures, especially in later pregnancy, and the development and implementation of novel tests (biomarkers) in the mother or in early pregnancy, help prevent stillbirth?” [13]. In addition, it would provide further evidence to guide the management of RFM with the ultimate aim of reducing the rate of stillbirth in line with national ambition while reducing unnecessary obstetric intervention [3].

## Supplementary information

**Additional file 1.** CONSORT 2010 checklist of information to include when reporting a pilot or feasibility trial.

**Additional file 2.** Trial recruitment by site.

**Additional file 3.** Scatter plot of site and central lab sFlt-1/PlGF ratio for participants in the intervention arm.

## Data Availability

The datasets used and/or analysed during this trial are available from the corresponding author on reasonable request.

## Abbreviations

AUROC: Area under the receiver operator characteristic curve
CI: Confidence interval
CONSORT: Consolidated Standards of Reporting Trials
CTG: Cardiotocograph
EFW: Estimated fetal weight
FASP: Fetal Anomalies Screening Programme
GAD-2: Generalised Anxiety Disorder 2
GROW: Gestation Related Optimal Weight
hPL: Human placental lactogen
ICU: Intensive care unit
IOL: Induction of labour
ISRCTN: International Standard Randomised Controlled Trial Number
NCTU: Nottingham Clinical Trials Unit
NHS: National Health Service
NICU: Neonatal intensive care unit
NIHR: National Institute for Health Research
PlGF: Placental growth factor
PPI: Patient and public involvement
ReMIT-2: Reduced fetal movement intervention-2 Trial
RFM: Reduced fetal movement
ROC: Receiver operator characteristic
SD: Standard deviation
sFlt-1: Soluble fms-like tyrosine kinase-1
SGA: Small for gestational age
