# Supplementary material for: Standard care informed by the result of a placental growth factor blood test versus standard care alone in women with reduced fetal movement at or after 36+0 weeks’ gestation: a pilot randomised controlled trial
Source: Pilot Feasibility Stud. 2020 Feb 13;6:23. doi: 10.1186/s40814-020-0561-z (PMC7020549; doi:10.1186/s40814-020-0561-z)
Supplement: Supplementary file 2 — Additional file 2. Trial recruitment by site. [file 40814_2020_561_MOESM2_ESM.docx]

Additional File 2: Trial recruitment by site

| **Site (length of time open to recruitment)** | **Number of live births per year (2015)^1^** | **Potentially eligible women during the trial period^2^** | **Not approached** | **Women approached** | **Approached but not consented^3^** | **Women consented and randomised^3^** |
| --- | --- | --- | --- | --- | --- | --- |
| St Mary’s Hospital, Manchester  (open 10 months) | 8, 934 | 529 | 459 (87%) | 70 (13%) | 12 (17%) | 58 (83%) |
| John Radcliffe Hospital, Oxford  (open 8 months) | 6,577 | 238 | 195 (82%) | 43 (18%) | 16 (37%) | 27 (63%) |
| James Cook University Hospital, Middlesbrough  (open 9 months) | 4,781 | 466 | 423 (91%) | 43 (9%) | 13 (30%) | 30 (70%) |
| Royal Preston Hospital  (open 9 months) | 4,320 | 595 | 548 (92%) | 47 (8%) | 25 (53%) | 22 (47%) |
| University Hospital of North Tees  (open 6 months) | 3,045 | 195 | 133 (68%) | 62 (32%) | 26 (42%) | 36 (58%) |
| Liverpool Women’s Hospital  (open 6 months) | 8,366 | 576 | 549 (95%) | 27 (5%) | 10 (37%) | 17 (63%) |
| St George’s Hospital, Tooting  (open 6 months) | 5,087 | 175 | 149 (85%) | 26 (15%) | 14 (54%) | 12 (46%) |
| Sunderland Royal Hospital  (open 5 months) | 3,099 | 143 | 109 (76%) | 34 (24%) | 20 (59%) | 14 (41%) |
| Total |  | 2917 | 2565 (88%) | 352 (12%) | 136 (39%) | 216 (61%) |

^[1](https://www.ons.gov.uk/file?uri=/aboutus/transparencyandgovernance/freedomofinformationfoi/hospitalstatistics/livebirthsbyce2015final.xls)^[https://www.ons.gov.uk › file › hospitalstatistics › livebirthsbyce2015final](https://www.ons.gov.uk/file?uri=/aboutus/transparencyandgovernance/freedomofinformationfoi/hospitalstatistics/livebirthsbyce2015final.xls)

^2^ Potentially eligible women were those who presented with RFM ≥36 weeks’ gestation

^3^Women approached is the denominator used for these columns
