# Supplementary material for: Standard care informed by the result of a placental growth factor blood test versus standard care alone in women with reduced fetal movement at or after 36+0 weeks’ gestation: a pilot randomised controlled trial
Source: Pilot Feasibility Stud. 2020 Feb 13;6:23. doi: 10.1186/s40814-020-0561-z (PMC7020549; doi:10.1186/s40814-020-0561-z)
Supplement: Supplementary file 3 — Additional file 3. Scatter plot of site and central lab sFlt-1/PlGF ratio for participants in the intervention arm. [file 40814_2020_561_MOESM3_ESM.docx]

Additional File 3: Scatter plot of site and central lab sFlt-1/PlGF ratio for participants in the intervention arm

**
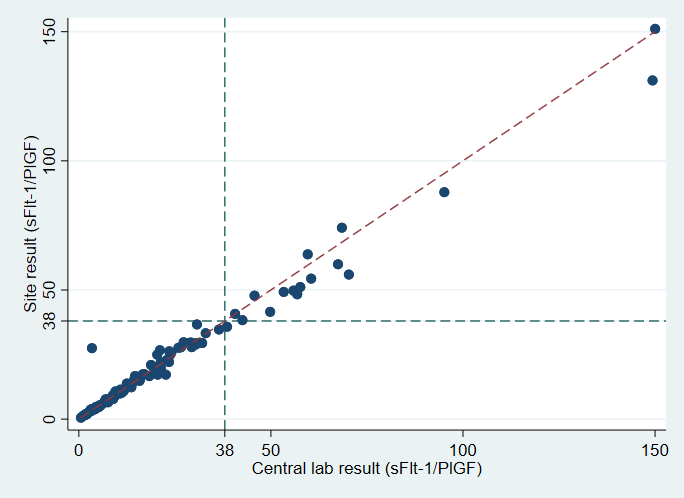
**
